# Cerebrovascular implications of takayasu arteritis: a review

**DOI:** 10.1007/s00234-024-03472-2

**Published:** 2024-10-08

**Authors:** Mena Samaan, Arevik Abramyan, Srihari Sundararajan, Emad Nourollah-Zadeh, Hai Sun, Anil Nanda, Sudipta Roychowdhury, Gaurav Gupta

**Affiliations:** 1 Department of Neurosurgery, Cerebrovascular and Endovascular Neurosurgery, Rutgers RWJ Barnabas Healthcare System, Rutgers Robert Wood Johnson Medical School, 10 Plum Street, 5 Floor, # 548, New Brunswick, NJ 08903-2601 USA; 2 Department of Interventional Neuroradiology, Robert Wood Johnson Medical School, University Radiology, New Brunswick, NJ USA; 3 Department of Neurology, Rutgers Robert Wood Johnson Medical School, New Brunswick, NJ USA

**Keywords:** Cerebrovascular, Intracranial, Ischemia, Takayasu, Vasculitis

## Abstract

**Purpose:**

Takayasu arteritis (TA) is a rare, chronic, inflammatory large-vessel vasculitis that affects the aorta and its main branches, including the cerebrovascular system. This review analyzes current knowledge and patient outcomes concerning the cerebrovascular implications of TA.

**Methods:**

A literature search, with publications from 1994 to 2024, identified pertinent studies through PubMed. An illustrative case report details a 19-year-old female with Type 1 TA, illustrating the complex decision required in the absence of surgical or endovascular options.

**Results:**

Our results offer a demographic analysis of 1,698 TA patients, highlighting a female predominance of 89.99% and a mean symptom onset at 33 years. The clinical spectrum of cerebrovascular involvement presented varied symptoms, most notably dizziness, with significant incidences of ischemic events and bilateral stenosis primarily affecting the carotid and subclavian arteries. The most common type of TA was Type V, affecting 40% of patients studied. Endovascular treatment had a 95% initial success rate, with a 67% restenosis rate. Surgical treatment was successful in 84% of cases, but 21% had notable post-operative complications. Similar to the endovascular population, those treated with stand-alone conservative therapy saw a 93% initial remission rate with 52% having relapsed.

**Conclusion:**

Assessing the disease activity of TA is crucial when planning vascular intervention due to its significant impact on treatment outcomes. Despite its greater initial invasiveness, surgical interventions showed lower restenosis rates compared to either endovascular interventions or standalone conservative management. We emphasize advancements in TA management and the pressing need for continued research into diagnostic and treatment protocols for improved patient outcomes.

## Introduction

Takayasu arteritis (TA) is a rare form of vasculitis, primarily affecting young women, that causes inflammation of large vessels, particularly the aorta and its main branches. Given its quick onset and nonspecific symptoms, TA poses diagnostic challenges, highlighting the necessity for clinical dexterity and proficiency in neurovascular imaging techniques for early detection and intervention. In this study, we performed a systematic review and presented a detailed case from our institution, aiming to enhance the understanding of TA's cerebrovascular implications and inform on the efficacy of various treatment modalities.

## Materials & methods

### Literature search strategy

A systematic literature review was performed in order to identify and analyze the incidence of cerebrovascular involvement and possible treatment modalities, which was done in accordance with the Preferred Reporting Items for Systematic Reviews and Meta-Analysis (PRISMA) checklist [1]. We performed an English literature search using the PubMed database with the keywords “Takayasu” and “intracranial” or “cerebr*. Additional studies were identified by examining references cited within selected articles. There were no search restrictions based on the type of study or language at this stage.

### Study selection and inclusion criteria

Two independent researchers (M.S. and A.A) examined all of the sources related to the search terms and formed the following inclusion criteria: (1) published studies reporting on TA with a focus on intracranial involvement and revascularization techniques, (2) articles that provide clear demographic data on patients, including age and sex, (3) studies detailing the clinical presentation and specific cerebrovascular features of TA, such as involvement of carotid, vertebral, or subclavian arteries, (4) publications that outline the treatment modalities employed, ranging from conservative management to surgical and endovascular interventions, (5) reports that include outcomes following treatment, including success rates, postoperative complications, and mortality data, (6) case series and reports that present individual or aggregated patient data relevant to the management of cerebrovascular aspects of TA, (7) full-text articles, with abstracts considered if they contain comprehensive information pertinent to the study's focus. Exclusion criteria included letters, unpublished articles, and cases of solely systemic implications.

### Data collection

**Fig. 1:**
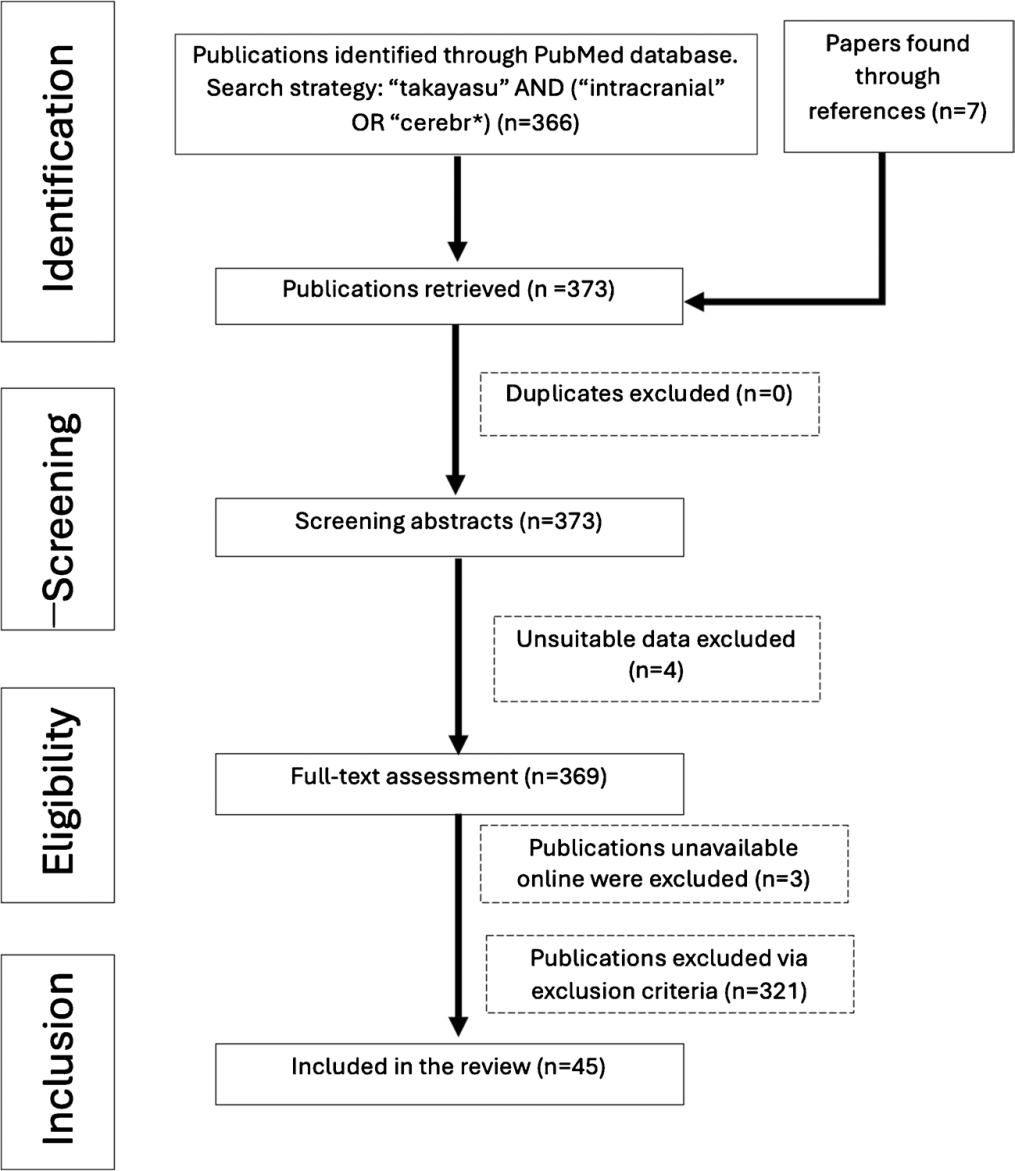
PRISMA-Based Flow Diagram of the Systematic Review

The following data were extracted and reviewed by two independent authors (M.S. and A.A): number of cases per paper, age and sex of participants, clinical symptoms and characteristics, imaging modalities and findings, rate and extent of systemic or cerebrovascular implications, treatment modalities, outcomes, and follow-up durations. This process aimed to mitigate potential extraction bias. The literature search strategy is visualized in Fig. 1.

## Results

### Search results and selected studies

Based on the inclusion and exclusion criteria, a total of 373 eligible articles were identified and reviewed, and the year of publication ranged from 1994 to 2024. The final bibliography is based on originality and relevance to the subject. There were no duplicates and the abstracts and headings of all 373 articles were screened via the inclusion criteria. A total of 328 studies were found ineligible, and a total of 45 studies were selected. Full text was available for all 45 studies, and no abstracts were included.

The three countries with the highest prevalence of cases included in this study were Canada (482 cases), China (399 cases), and France (336 cases). The clinical presentations and outcomes of cerebrovascular implications in TA are summarized in Table 1. An additional summary of published cases of TA accompanied by Moyamoya disease (MMD) is presented in the discussion.

**Table 1:** Clinical Presentation, Imaging Findings, Treatment Modalities, and Outcomes in Cerebrovascular Takayasu Arteritis Patients

Reference	*N* (patients)	Mean Age (years) / % Female	Clinical Symptoms	Imaging Findings	Conservative Management	Endovascular Repair	Surgery	Comparative Outcomes
Gao et al. 2020 [2]	1	59 / 100%	Hemiparesis, aphasia	Occlusion	100% remission	N/A	N/A	N/A
Field et al. 2017 [3]	1	52 / 100%	Hemiparesis, aphasia	Thrombosis	100% remission	N/A	N/A	N/A
Hoffman et al. 1994 [4]	18	30 / 83%	Claudication, fever, headache	Stenosis, occlusion, or aneurysm	81% remission with 44% relapse	N/A	N/A	N/A
Maksimowicz-McKinnon et al. 2007 [5]	30	27 / 97%	Claudication, hypertension, dizziness, visual disturbance	Stenosis or aneurysm	20% remission	90% success, 78% restenosis	68% success,12% post-operative deaths	Most effective: Surgery Lowest complication rate: Surgery
Comarmond et al. 2012 [6]	84	28.5 / 81%	Claudication	Stenosis	37% remission	N/A	N/A	N/A
El Hunjul et al. 2023 [7]	1	17 / 100%	Hemiparesis, syncope	Stenosis	N/A	N/A	100% success	N/A
Kim et al. 2012 [8]	25	34 / 88%	Visual disturbance, dizziness	Stenosis, occlusion, aneurysm, or TIA	N/A	100% success, 40% restenosis, 13% occlusion	100% success, 6% restenosis, 12% occlusion, 13% ICH	Most effective: Surgery Lowest complication rate: Surgery
Han et al. 2017 [9]	19	40.6 / 100%	Visual disturbance, syncope, headache	Stenosis or occlusion	N/A	N/A	69% success, 16% ICH, 16% peri-operative deaths	N/A
Johnson et al. 2021 [10]	20	29.5 / 90%	Claudication, visual disturbance, headache	Stenosis or occlusion	N/A	N/A	N/A	N/A
Couture et al. 2018 [11]	17	42 / 88%	Claudication, fever	Stenosis	N/A	N/A	N/A	N/A
Hiu et al. 2008 [12]	1	63 / 100%	Syncope, visual disturbance, dizziness	Occlusion	N/A	N/A	100% success	N/A
Tyagi et al. 2008 [13]	10	28.3 / 60%	Syncope, visual disturbance, seizure	Stenosis	N/A	100% success, 20% restenosis	N/A	N/A
El Mesnaoui et al. 2007 [14]	7	33.8 / 86%	Cerebral insufficiency symptoms	Thrombosis or occlusion	N/A	N/A	71% success, 43% thrombosis,14% peri-operative deaths	N/A
Luo et al. 2017 [15]	29	24 / 90%	Hemiparesis, syncope, visual disturbance, dizziness	Stenosis or occlusion	N/A	92% success, 68% restenosis	88% success, 53% CHS, 6% ischemic stroke	Most effective: Surgery Lowest complication rate: Surgery
Wang et al. 2006 [16]	103	27.6 / 89%	Dizziness, headache, visual disturbance, hemiplegia	Not specified	89% remission, 7% stroke, 7% deaths	100% success, 100% restenosis	87% success, 8% deaths	N/A
Gu and Wang 2001 [17]	49	26.5 / 88%	Dizziness, headache, visual disturbance, hemiplegia	Not specified	N/A	N/A	90% success, 7% restenosis, 10% peri-operative deaths	N/A
Zhang et al. 2011 [18]	1	12 / 0%	Syncope, seizure	Encephalomalacia and infarctions	100% remission, 100% relapse, 100% deaths	N/A	N/A	N/A
Skeik et al. 2013 [19]	1	29 / 0%	Hemiplegia	Thrombosis and stenosis	N/A	N/A	100% success	N/A
Bond et al. 2017 [20]	79	33.2 / 91%	Claudication, dizziness, headache, visual disturbance, syncope, hemiplegia	Stenosis and occlusion	N/A	N/A	N/A	N/A
Lee et al. 2018 [21]	1	14 / 100%	Hypertension	Encephalomalacia and stenosis	100% success	N/A	100% success	N/A
Li et al. 2021 [22]	1	27 / 100%	Visual disturbance, syncope	Occlusion	100% success	N/A	N/A	N/A
Beijk et al. 2023 [23]	1	52 / 100%	Dizziness, headache, visual disturbance	Occlusion	N/A	N/A	100% success	N/A
Uzunlar et al. 2014 [24]	1	27 / 100%	Syncope	Stenosis	N/A	N/A	N/A	N/A
Shuaib et al. 2013 [25]	1	21/ 100%	Hemiparesis, aphasia	Hemorrhage	100% success	N/A	N/A	N/A

### Demographics

According to our findings of 12 case reports and 22 case series describing 1,698 patient cases, demographic information was available for 1,575 (93%) patients. The gender distribution was 1,448 (89.99%) females to 161 (10.01%) males. The mean age of onset of symptoms was 33.27 ± 9.221 years. Ages ranged from 5 to 59 years old. Ethnicity was available for 867 out of 1,698 (51%) total patients, of which the top five ethnic groups consisted of 368 (42%) Chinese patients, 271 (31%) Caucasian patients, 83 (10%) North African/Arab patients, 64 (7%) Black patients, and 55 (6%) Korean patients.

### Clinical data

Clinical presentation varied, with 841 total symptoms reported; the most common symptoms were hypertension as 30% of symptoms, dizziness as 13%, visual disturbance as 11%, claudication as 10%, and fatigue and headache as 9% each. Of the 3,034 cranial arteries studied, the most common cerebrovascular anomalies involved 975 (32%) carotid arteries, 933 (31%) subclavian arteries, and 131 (4%) vertebral arteries. There was bilateral stenosis in 328 of the 804 patients (41%) with TA-related cerebrovascular involvement. Of the 955 lesions histologically analyzed, 582 (61%) were active lesions, characterized by perivascular mononuclear cell infiltrates in the vasa vasorum. The most common type of TA based on our analysis of 428 patients was Type V affecting 172 (40%) patients, followed by Type I affecting 134 (31%) patients, Type IV affecting 41 (10%) patients, Type IIa affecting 33 (8%) patients, Type IIb affecting 30 (7%) patients, and Type 3 affecting 18 (4%) patients.

Treatment outcomes were available for 1,391 therapies or procedures for those with cerebrovascular TA; of those, a total of 711 (51%) patients underwent standalone conservative therapy, while 516 (37%) underwent surgical treatment, and 164 (12%) of patients underwent endovascular treatment. Endovascular treatment was initially successful in 155 (95%) cases with restenosis in 104 (67%) of those cases. No major intra- or peri-procedural complications were reported for the endovascular group. Surgical treatment resulted in complete resolution in 435 (84%) cases. Of the successful surgeries, 48 (9%) patients died, 27 (5%) patients had cerebral hyperperfusion syndrome (CHS), 15 (3%) patients had thrombosis/occlusion, 12 (2%) patients had stenosis, and 9 (2%) patients had stroke. Standalone conservative therapy resulted in remission in 342 (48%) cases, with relapse in 73 (10%) of those cases. In this group, there were 2 (3%) strokes and 3 (4%) deaths. Follow-up was available for 430 (60%) patients treated with conservative management, of which 401 (93%) patients sustained initial remission and 223 (52%) patients had at least one relapse during tapering.

These findings are presented in Table 1

## Illustrative case report

A 19-year-old female presented with acute aphasia, right-sided hemiparesis, disorientation, unresponsiveness to verbal commands, diminished pedal pulses, and breakthrough seizures. She had a history of Type 1 TA, aortic stenosis and regurgitation, seizure disorder, and a right middle cerebral artery (MCA) stroke three years prior to arrival, in which she underwent a right-sided hemicraniectomy and sustained left-sided hemiparesis. The patient was able to follow commands but was not communicating verbally and had multiple episodes of convulsions without intervention.

**Fig. 2:**
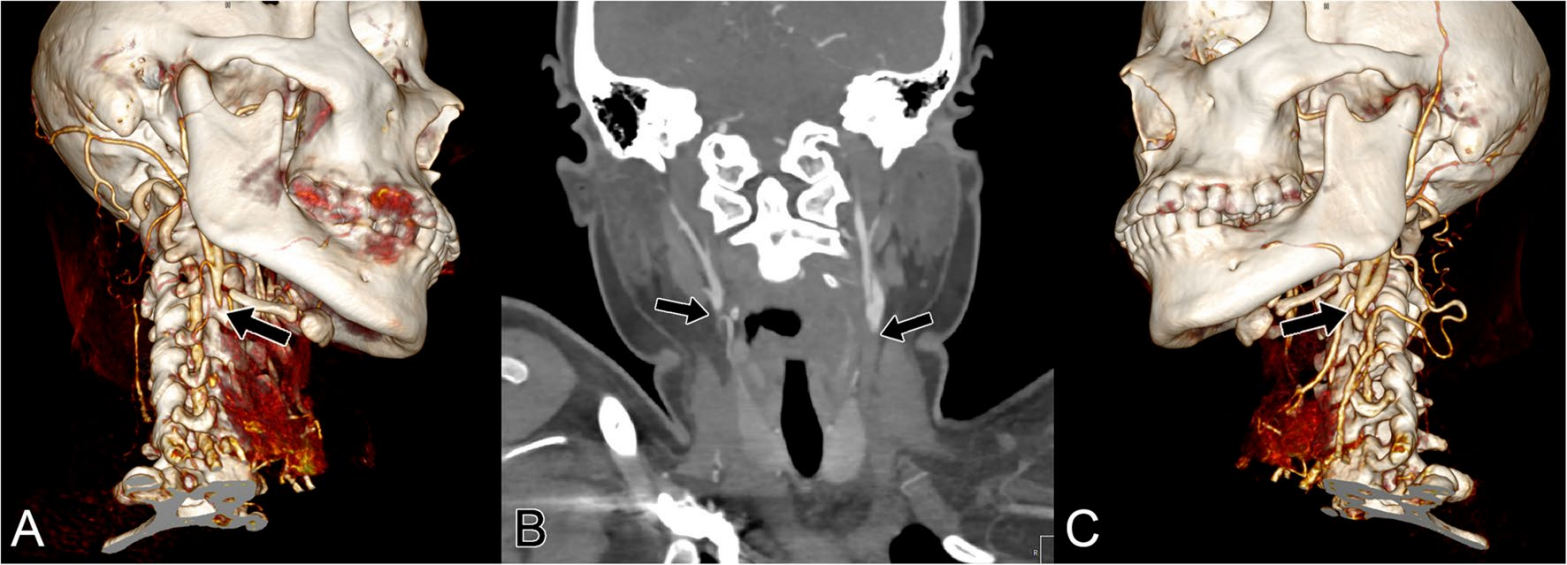
Neck CTA showing bilateral CCA occlusion (black arrows). Figure 3A and 3C: CTA 3D reconstruction. Figure 3B: MIP imaging modality

**Fig. 3:**
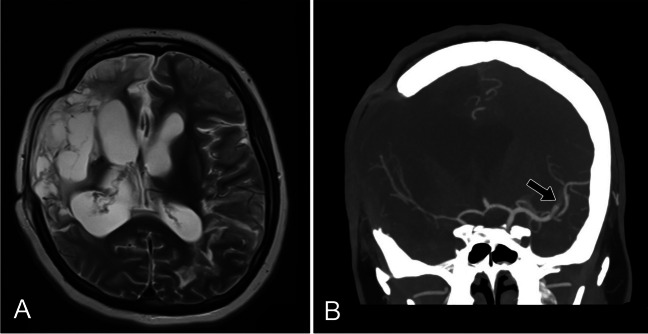
A MRI T2 showing encephalomalacia, B CTA MIP showing thromboembolic occlusion (black arrow) of the M2 branch of the left MCA

Computed tomography angiography (CTA) of the head and neck, and computed tomography (CT) head perfusion studies were performed (Fig. 2). The imaging revealed occlusion of the right internal carotid artery (ICA), both common carotid arteries (CCA), the left vertebral artery, the left M2 branch, and the left distal M1 branch, as well as a MCA thrombus with filling defect, and a left proximal ICA thrombus (Fig. 3). The studies also showed encephalomalacic right frontal lobe bulges through the craniectomy site, with 7 mm left-to-right midline shift due to negative mass effect, along with moderate ex vacuo dilatation of the bilateral lateral ventricles. There was evidence of previous reconstitution to the distal inferior M3 branches, the supraclinoid carotid artery, the right ophthalmic artery, the right CCA, and the distal V1 branch.

After a multi-disciplinary meeting with neurology, rheumatology, radiology, and intensive care was held, it was deemed that the patient was outside the window of tissue plasminogen activator (tPA) administration, and that mechanical thrombectomy (MT) was unable to be performed due to excessive CCA occlusion. The decision was made to continue with conservative management with prolonged high dose steroids.

One week later, a repeat magnetic resonance angiography (MRA) of the brain showed multiterritory infarcts involving the left MCA, a punctate infarct in the left anterior cerebral artery (ACA), and small left posterior cerebral artery territory infarcts with trace subarachnoid hemorrhage. One month after admission, the patient was able to ambulate with a cane and conversed normally but had sustained left-sided hemiparesis. MRA revealed reconstitution of flow to the previously noted left superior M2 division occlusion.

Two months after admission, repeat MRI/MRA brain scans appeared mostly unchanged, despite aggressive immunosuppression with prolonged high dose steroids. It was determined that her imaging findings represented chronic stenosis, as opposed to active inflammation. The decision was made to taper the patient off the corticosteroids. The decision was communicated with the family, who would like to continue physical and occupational therapy with hope for improvement or recovery.

## Discussion

### Incidence

TA is a rare and chronic inflammatory disease characterized by granulomatous inflammation of the aorta and its major branches, as well as the pulmonary, renal, brachiocephalic, common carotid, and subclavian arteries [2]. Our findings affirm the demographic trends noted in the existing literature that TA primarily affects young women. Geographical variations in the prevalence of TA have been recorded, with higher numbers reported in Asian populations. The incidence of TA varies geographically, with estimates ranging from 1–2 per million in Japan to 0.9 per million in the US [26]. The highest ever prevalence was observed in Japan, estimated to be 40 per million [27]. As we found a high prevalence of Asian populations, the ethnic distribution from our review aligns with that typically described in the literature.

### Etiology

Stenosis in TA is primarily the result of a complex interplay between inflammation, immune response, and vascular remodeling. TA typically progresses through a systemic inflammatory stage and a vascular manifestation stage. In the first stage, the arterial wall engages with activated T cells and macrophages, leading to luminal stenosis or aneurysmal wall damage of the vessel [28]. The second stage is characterized by adventitial fibrosis, intimal smooth muscle proliferation, and vessel stenosis [29]. Although the exact cause of the autoimmune response component of TA is not yet fully understood, it is thought to be associated with genes encoding human leukocyte antigen class I and class II specificities, immune response regulators, and proinflammatory cytokines [30]. Thaunat et al. suggest a role for the neoangiogenic vasa vasorum as conduits for the entry of inflammatory cells into the graft [31]. After inflammatory cells accumulate and several cytokines are released, the existing vasa vasorum is stimulated to expand and form new, highly permeable vessels, further exacerbating the number of inflammatory cells [32]. The progressive accumulation of leukocytes in the intima, which triggers proliferation of smooth muscle cells in the media, results in vessel wall thickening and hypoxia. This continuous inflammatory stimulation can lead to an irreversible change in endothelial cells, resulting in a consistent migratory and proangiogenic state [33].

### Clinical manifestations

The main clinical manifestation of TA is the presence of vascular bruits. One review analyzing over 570 patients from different countries found diminished or absent pulses and the presence of vascular bruits in around 90% of patients with TA [34]. The next most common clinical findings were hypertension, renal artery stenosis, Takayasu retinopathy, and pulmonary artery involvement. In our analysis, hypertension, dizziness, and visual disturbances emerged as the most common clinical symptoms, aligning with traditional TA presentations and emphasizing the disease's systemic impact.

### Classification

TA is classified according to the anatomical distribution of the vasculitis, the type of vessel involvement, and the disease's progression. The traditional classification divides TA into Types I-V, with each type corresponding to particular regions of the arterial system, as detailed by the Numano classification system [35]. Type I involves the branches of the aortic arch. Type IIa includes the ascending aorta, aortic arch, and its branches, while Type IIb extends to the thoracic descending aorta. Type III affects the thoracic descending aorta, abdominal aorta, and/or renal arteries. Type IV involves the abdominal aorta and/or renal arteries. Type V combines features of types IIb and IV, affecting the ascending aorta, aortic arch, its branches, thoracic descending aorta, abdominal aorta, and/or renal arteries. A more recent classification system also considers the vessel wall's morphological changes as seen through imaging modalities, which considers the patterns of stenosis, occlusion, dilatation, and aneurysm formation. The early, pre-pulseless phase of the disease, characterized by systemic inflammation, and the late, pulseless phase, with its consequent vascular stenosis or occlusion, are essential for understanding the disease progression and potential complications. These classification systems are crucial for directing the therapeutic approach and assessing the prognosis for individuals with TA.

### Diagnosis

The diagnosis of TA remains complex, requiring the integration of clinical assessment and imaging modalities to analyze the characteristic features of this large vessel vasculitis. According to Furuta et al., differentiation from other conditions like giant cell arteritis is critical and often relies on radiological findings that distinguish TA, such as wall thickening, vessel stenosis, and the presence of aneurysms or occlusions in the aorta and its main branches [36]. In terms of imaging modalities, digital subtraction angiography (DSA) is deemed the gold-standard. Nevertheless, in a comparison of accuracy between DSA and CTA in 25 TA patients, the sensitivity and specificity of CTA in the diagnosis of TA was noted to be 95% and 100%, as compared to DSA’s 93% and 98%, respectively [37]. Duarte et al. and Mirouse et al. provide evidence via meta-analyses supporting the association of TA with cerebrovascular ischemic events, which can often prompt diagnostic investigation in otherwise asymptomatic patients [38, 39].

### Ischemic stroke in takayasu arteritis

While the stroke rate in patients with TA is approximately 15–20%, studies indicate that the prevalence of cerebrovascular implications is notably higher, with one review observing a range from 31% to 45%  [38, 40] Pediatric cases exhibit a particularly high incidence, with reports suggesting cerebrovascular implications in 42.9%-71% of patients [41]. One study of 23 TA patients found that 52% of their population had carotid artery stenosis, 30% had brachiocephalic artery stenosis, and 13% had vertebral artery stenosis [36]. Another study of 461 TA patients found the left carotid artery involvement and left subclavian artery involvement to be 46% and 56%, respectively [42]. Similarly, involvement of the CCA is shown through diffuse or circumferential thickening of the vessel wall, which is significantly thicker in active than in inactive lesions [43]. The steno-occlusive changes to the cerebral perfusion and its associated neurological complications must be recognized early in this patient population, so as to avoid further occlusion or ischemic stroke.

Ischemic stroke in TA is attributed to either occlusive compression or embolic blockages. The literature reports varying rates of stroke occurrence in TA, and a recent meta-analysis by Mirouse et al. reported a stroke or transient ischemic attack (TIA) rate of 20%, which aligns with a rate of 15.8% from Duarte et al.’s previous meta-analysis [30, 31]. Field et al. describe a case of a Caucasian woman who presented with signs of acute ischemic stroke, who was treated with administration of tPA and had a repeat stroke two days after discharge [3]. The authors reported that patients with active disease who underwent operations were more likely to develop thrombosis or restenosis. In their case, MT was deferred due to concerns related to the friability of the vessel in the setting of severe inflammation. In our case, MT was also considered but ultimately could not be performed due to compete occlusion of both CCAs. As such, the disease activity at the time of intervention is an important factor to consider when planning vascular intervention. The case by Field et al. attributes the stroke to arterial stasis secondary to compression of the vessels from vasculitis, indicating a direct link between TA-induced vascular changes and stroke. In our case, the extensive vascular involvement and occlusions also suggest a complex interplay of stenosis, occlusion, and embolism as contributing factors to the stroke. Reflecting on our systematic review findings, cerebrovascular complications were notably prevalent, with bilateral stenosis identified in 41% of patients with cerebrovascular involvement, highlighting the critical importance of timely and accurate vascular intervention.

### Conservative management

In terms of treatment strategies for TA patients, antiplatelet and anticoagulant agents aim to prevent further occlusion by controlling active thrombosis [2]. Starting an immunosuppressive regimen early is indicated to prevent further vasculopathy. Methotrexate and azathioprine are two agents that are thought to stop the progression of arterial lesions [4]. However, in one retrospective study of 75 patients with TA treated with either immunosuppressive agents or vascular intervention, only 28% of patients sustained long-term remission with the standalone use of the corticosteroid prednisone, while 17% sustained long-term remission after tapering to discontinuation [5]. Similarly, our patient was placed on methotrexate at the time of diagnosis 3 years ago, and has not since shown resolution of arterial lesions. In another study by Perera et al., biopsy data revealed active inflammation in 58.3% of patients thought to be in clinical remission [44]. In a nationwide, retrospective multicenter study, most patients treated with standalone glucocorticosteroids achieved initial disease remission [45]. However, relapse was seen in more than half of the patients during tapering to discontinuation of the corticosteroids. It is also important to note that chronic glucocorticosteroids therapy has associated secondary pathologies, including diabetes, hypertension, cardiovascular disease, and infection.

Another class of medication with functions applicable towards treatment in TA are monoclonal antibodies, including tumor necrosis factor-α (TNF-α) inhibitors like infliximab and etanercept. In one study of 84 TA patients treated with TNF-α inhibitors, complete remission was seen in 36% of patients, partial remission in 54% of patients, and no response in 10% of patients [6]. The IL-6 receptor inhibitor tocilizumab, the B-cell depleting agent rituximab, the immunomodulator leflunomide, and other monoclonal antibodies have all shown to be effective in remission maintenance, glucocorticoid-sparing, radiographic response, and cost-effectiveness [46]. From our review, the initial remission rate with standalone conservative management was found to be high, at 93%; however, more than half of these patients (52%) relapsed during tapering, highlighting the complexities of managing TA with conservative treatment alone.

### Cerebrovascular endovascular vs surgical treatment

While systemic implications of TA often necessitate diverse treatment strategies, our review focuses on cerebrovascular pathology treatments.

Surgical treatments mainly consist of bypass procedures, linking vessels normal on angiography proximal and distal to the occlusive or stenotic lesion, while the endovascular interventions available consist of angioplasty, MT, and stenting [8, 47]. Although the indication for surgical intervention is typically held at a stenosis rate of 70%, the potential benefits and drawbacks must be carefully examined [7]. In a similar case to ours, a Hispanic teenaged female with TA presented with severe stenosis of the left proximal, right proximal, and mid-left CCAs [7]. After medical management with immunologic therapy and warfarin was attempted and then deemed insufficient, surgical management was pursued. The patient underwent an aorta-right-CCA bypass with a vascular tube graft, and had notable improvement one month later [43]. As lesions to the CCAs or subclavian arteries are often long, irregularly fibrosed, and stenotic, angioplasty and stenting will be unsuccessful in most cases [44]. According to our data, surgical interventions were necessary in 37% of cases, with bypass surgery showing a higher success rate and a lower incidence of restenosis at 9%, compared to 67% in endovascular treatments. This advocates for a more strategic approach in the management of systemic implications.

Extra vigilance and awareness are needed to mitigate complications associated with these surgical procedures. Han et al. report on the outcomes and complications of aorto-carotid bypass procedures, in which they had an intracranial hemorrhage rate of 26%, intracranial infarction and CHS rates of 21% each, and graft occlusion and mortality rates of 16% each [9]. Similarly, endovascular intervention also has troubling complications reported in the literature. For example, Cong et al. report that the restenosis associated with endovascular treatment can often be traced back to the extensive, fibrotic nature of vessel lesions characteristic of TA and the trauma induced by dilatation, which leads to myointimal proliferation [48]. This restenosis presents a challenge, as it often requires additional interventions, highlighting the need for a more durable initial treatment protocol. Additionally, the decision for revascularization should be multidimensional, informed not only by radiologic imaging but also by comprehensive assessments of neurological symptoms, as these may help reveal the development of collateral circulation, which could influence the course of treatment [48].

In assessing the clinical decision-making for severe cerebral ischemia in TA, our study draws upon insights from Luo et al. who report that surgical interventions may be imperative even during the active phase of TA, especially when ischemic symptoms rapidly exacerbate despite pharmacological treatment [15]. Other studies have acknowledged the potential dangers of surgical intervention during the active phase of TA, but further emphasize the critical need for such procedures in certain clinical scenarios in which severe symptoms persist or escalate despite conservative measures [8, 9, 44]. In circumstances of localized involvement, or when surgical risks were deemed too great, endovascular treatments were considered as a viable alternative. Luo et al. also highlight the necessity of careful postoperative management to mitigate CHS, a notable risk factor for perioperative mortality [15]. Importantly, this highlights the necessary role of blood pressure control, mannitol administration, and routine transcranial Doppler ultrasound to monitor for CHS, with a significant increase in blood flow velocity serving as a critical indicator.

Endovascular interventions for focal lesions, despite a higher incidence of restenosis, were recognized for their initial symptom relief, highlighting the potential for collateral vessel formation. Such insights are particularly relevant to the course of treatment for TA patients with more nuanced needs, such as those at both extremes of age, to achieve the dual goals of symptom management and long-term vascular patency.

As per our results, the comparative analysis between cerebrovascular surgeries, endovascular treatments, and conservative management has yielded a distinct preference towards bypass surgery for resolving stenotic cerebrovascular complications in TA patients, as evidenced by the substantial lower restenosis rates when compared to endovascular interventions or standalone conservative management. Surgical interventions, specifically bypass procedures, have shown a higher success rate in terms of symptom resolution and long-term vessel patency. Moreover, our data indicate that while the immediate relief from endovascular procedures is noteworthy, the restenosis rate necessitates a consideration of the long-term objectives. The enduring effectiveness of bypass surgery, despite its greater initial invasiveness, offers sustained improvement of cerebral ischemic symptoms.

Conservatively managed patients, while avoiding the risks associated with surgical interventions, faced progression in symptomatology and a higher incidence of subsequent invasive procedures. This progression highlights the need for a proactive treatment strategy in certain patient demographics, particularly those with rapidly progressing disease or critical stenosis. As such, it appears as if bypass surgery is the most curative treatment option for TA patients with extensively stenotic cerebrovasculature. This is not to discount the role of conservative and endovascular treatments, which remain valuable for patients with contraindications to surgery, or as part of a multi-faceted treatment strategy. Our results advocate for a personalized treatment protocol, considering individual patient risk profiles, the extent of vascular involvement, and the dynamic nature of TA progression.

### Moyamoya disease and takayasu arteritis

The incidence of MMD and TA together is extremely low. However, analyzing the correlations between these two diseases can provide insight into novel treatment strategies [18–21]. Currently, only 4 cases of concurrent MMD and TA exist in the literature, and 3 of these 4 cases noted that MMD manifested secondary to TA [18, 19, 21]. The literature already notes that MMD likely requires a secondary “trigger” to induce the mutated genes associated with the disease to begin expression [49]. As such, it is possible that TA can serve as this trigger. All reported patients were initially treated for the TA’s systemic implications with either corticosteroids or renal angioplasty, and all patients re-presented with new cerebrovascular infarcts or stenoses, along with the formation of collateral vessels. These cases highlight an important distinction between conservative management and more aggressive approaches when determining the course of treatment in these patient populations. In the one case where the patient was only treated with further corticosteroids, the patient re-presented 2 weeks later with multiple large infarcts, encephalomalacia, new cerebrovascular occlusions, and a foramen magnum herniation [16]. The patient then died after a failed rescue procedure attempt. In the other two cases where surgical intervention was performed, left superficial temporal artery to MCA bypasses with encephalomyosynangiosis were successful in stabilizing the patients, and ultimately reversed their symptoms. It is crucial to note, however, that in one of these cases, the patient initially underwent a left-to-right carotid-carotid bypass which was not curative, and the other patient later underwent a right encephaloduromyosynangiosis [17, 19]. With the consideration of overlapping pathologies, these cases enable interventionists to approach cases of cerebrovascular TA with different treatment approaches. Despite the apparent success of these procedures, extreme caution must be taken when analyzing their efficacy in TA patients, as they may cause postoperative cerebral watershed shift or CHS, namely due to the accumulation of pro-inflammatory cytokines [21]. Notably, the improvement of cerebral perfusion after revascularization may expose the cerebral deep watershed zone to ischemia or CHS via the Venturi effect, the phenomenon where an increase in blood flow velocity after surgical revascularization inadvertently reduces perfusion to surrounding cerebral tissues [21].

### Limitations

The limitations of this systematic review primarily stem from the varied methodological quality and reporting standards of the included studies, which may introduce heterogeneity and affect the consistency of our findings. The rarity of TA limits the available literature, and consequently the depth of data that can be analyzed, which potentially affects the generalizability of our results. Despite the systematic and comprehensive nature of our search, publication bias and the potential omission of unpublished or non-English studies could influence our conclusions. Moreover, the dynamic and multifaceted nature of TA treatment complicates the aggregation of data across different treatment modalities and geographic regions. Prospective studies with uniform diagnostic criteria and standardized treatment protocols would be valuable to confirm our findings and provide more definitive guidance for the management of this complex condition.

## Conclusion

Although TA remains an idiopathic disease with a complex pathogenesis, recent advancements have been made in our understanding of its etiology, progression, and treatment modalities. Similarly, there are newer biologic therapies that appear highly promising, such as monoclonal antibody therapies. The consideration of disease activity is an important factor that establishes all aspects of condition management, particularly when planning vascular interventions. Despite these advancements, challenges persist in assessing disease activity, establishing accurate diagnostic criteria, and deciding the appropriate approach among conservative management, endovascular repair, and surgery. As this area of research grows, a more nuanced understanding of TA will facilitate better patient outcomes, tailored treatments, and improved quality of life for those affected by this disease.

## Declarations

### Final approval of the version to be published

All authors approved the final version to be published.
